# Dual pharmacological inhibition of glutathione and thioredoxin systems synergizes to kill colorectal carcinoma stem cells

**DOI:** 10.1002/cam4.844

**Published:** 2016-08-03

**Authors:** Genki Tanaka, Ken‐ichi Inoue, Takayuki Shimizu, Kazumi Akimoto, Keiichi Kubota

**Affiliations:** ^1^ Department of Second SurgeryDokkyo Medical UniversityShimotsuga‐gunTochigi‐ken321‐0293Japan; ^2^ Center for Research SupportDokkyo Medial UniversityShimotsuga‐gunTochigi‐ken321‐0293Japan

**Keywords:** colorectal carcinoma, glutathione, NRF2, pentose phosphate pathway, thioredoxin

## Abstract

NRF2 stabilizes redox potential through genes for glutathione and thioredoxin antioxidant systems. Whether blockade of glutathione and thioredoxin is useful in eliminating cancer stem cells remain unknown. We used xenografts derived from colorectal carcinoma patients to investigate the pharmacological inhibition of glutathione and thioredoxin systems. Higher expression of five glutathione S‐transferase isoforms (*GSTA1, A2, M4, O2, and P1*) was observed in xenograft‐derived spheroids than in fibroblasts. Piperlongumine (2.5–10 *μ*mol/L) and auranofin (0.25–4 *μ*mol/L) were used to inhibit glutathione S‐transferase *π* and thioredoxin reductase, respectively. Piperlongumine or auranofin alone up‐regulated the expression of NRF2 target genes, but not TP53 targets. While piperlongumine showed modest cancer‐specific cell killing (IC_50_ difference between cancer spheroids and fibroblasts: *P* = 0.052), auranofin appeared more toxic to fibroblasts (IC_50_ difference between cancer spheroids and fibroblasts: *P* = 0.002). The synergism of dual inhibition was evaluated by determining the Combination Index, based on the number of surviving cells with combination treatments. Molar ratios indicated synergism in cancer spheroids, but not in fibroblasts: (auranofin:piperlongumine) = 2:5, 1:5, 1:10, and 1:20. Cancer‐specific cell killing was achieved at the following drug concentrations (auranofin:piperlongumine): 0.25:2.5 *μ*mol/L, 0.5:2.5 *μ*mol/L, or 0.25:5 *μ*mol/L. The dual inhibition successfully decreased CD44v9 surface presentation and delayed tumor emergence in nude mouse. However, a small subpopulation persistently survived and accumulated phosphorylated histone H2A. Such “persisters” still retained lesser but significant tumorigenicity. Thus, dual inhibition of glutathione S‐transferase *π* and thioredoxin reductase could be a feasible option for decreasing the tumor mass and CD44v9‐positive fraction by disrupting redox regulation.

## Introduction

Despite significant advancements in cancer therapeutics, residual disease and pan‐resistance (i.e., against any available chemotherapies and radiotherapy) remain a persistent problem in many patients 1. The concept of cancer stem cells (CSCs, also termed cancer‐initiating cells) was proposed as a responsible factor for residual disease 2, 3. CD44v9, one of the best‐characterized surface antigens of CSCs, interacts with cystine/glutamate transporter for cystine uptake 4, 5. A continual cystine supply is crucial for *de novo* synthesis of glutathione and thioredoxin antioxidant peptides, highlighting CD44v9's role in redox regulation 4, 5. The transcription factor NRF2 (encoded by the *NFE2L2* gene), a master regulator against oxidative stress, shows tumor‐specific nuclear localization as well as phosphorylation in surgical specimens of hepatocellular carcinoma (HCC) 6. It activates a battery of antioxidant genes classified into three subgroups: glutathione system, thioredoxin system, and NADPH production 7, 8, 9. Of note, levels of the enzymes of the pentose phosphate pathway (PPP), a major source of NADPH, increase in surgical specimens of colorectal carcinomas (CRCs) 10. The significance of augmented NADPH production is in the recycling of glutathione and thioredoxin antioxidant peptides (Fig. S1). Thus, both glutathione and thioredoxin depend on NADPH's reducing power to continually recycle their antioxidant functions (Fig. S1). NRF2, as a redox sensor and feedback regulator, integrates three groups of enzymes (those associated with the glutathione and thioredoxin systems and NADPH production) to reduce reactive oxygen species (ROS) 9. Hence, NRF2 stabilizes intracellular redox potential and ensures robust cellular systems against potential harmful effects of ROS. For example, once the glutathione system is disrupted (e.g., by pharmacological inhibition), increased ROS activates NRF2, and subsequently, the other thioredoxin system is augmented for compensation. Indeed, combined inhibition of glutathione and thioredoxin systems synergizes to kill cancer cells 11, suggesting the existence of mutually compensatory mechanisms. Whether such a combination strategy is useful for eliminating CSCs, specifically to eradicate residual diseases, remains unknown. Accumulating evidence indicate that pharmacological inhibition against ROS protection system is indeed a promising anticancer strategy 12, 13. Importantly, such approach appears to be effective irrespective of TP53 status 12, 13, suggesting that elevating ROS could kill cancer cells with diverse array of mutational profiles.

In this study, we investigated the pharmacological inhibition against glutathione, thioredoxin, or PPP using CRC patient‐derived xenograft tumor cells. Pharmacological inhibition against glutathione S‐transferase (GST)*π* or thioredoxin reductase (TXNRD) up‐regulated the expression of NRF2 target genes, but not of TP53 target genes, in CRC spheroids. Dual inhibition of GST*π* and TXNRD synergistically caused cell death and the synergism was more remarkable in CRC spheroids than in normal fibroblasts. Dual inhibition successfully decreased CD44v9 surface presentation and delayed tumor emergence in nude mouse. A small subpopulation of CRC spheroids showed accumulated double‐strand DNA breaks but nevertheless retained lesser but significant tumorigenicity. Therefore, dual inhibition of GST*π* and TXNRD is a feasible option for decreasing the tumor burden and CD44v9‐positive fraction. However, this approach could potentially fail to eliminate residual disease.

## Materials and Methods

### Patients and biospecimens

This study was approved by the Institutional Review Board of Dokkyo Medical University Hospital (ID: 26015), on the basis of the Ethical Guidelines for Clinical Research of the Ministry of Health, Labor and Welfare, Japan. Patients who were diagnosed as having CRC at Dokkyo Medical University Hospital agreed to donate the surgically resected tumor specimens for research purposes.

### Tumor serial transplantation in nude mice

All experimental procedures were approved by the animal facility at Dokkyo Medical University (ID: 951) and were described elsewhere 10. The existence of CSCs has been experimentally proven through serial xenotransplantation and tumorigenesis 2, 14. In order to obtain a tractable model of colorectal CSCs, we subcutaneously xenotransplanted tumors derived from 72 CRC patients, among that 20 specimens formed tumors after 1–4 months. These subcutaneous tumors were again subjected to enzymatic dissociation and some of the tumor cells were subcutaneously transplanted into other mice. The tumor cells, containing a sufficient number of colorectal CSCs, were successfully transplanted more than three times in 12 cases. We confirmed that such tumors contained a substantial number of CD44v9‐positive cells (a known CSC marker 5, 15). CD44v9‐positive rate was stable for at least 1 week (data not shown), even after dissociated tumors had been cultured as spheroids *in vitro* (cancer tissue‐originated spheroids: CTOS). Therefore, we maintained patient‐derived xenografts *in vivo* and used primary culture of CTOSs as accessible model of colorectal CSCs. Seven cases (CTOS:40, 42, 68, 71, 76, 86, 88) were used for *in vitro* experiments.

### Cell culture

Modified procedures of CTOS culture 16 were described elsewhere 10. Enzymatically dissociated cancer cell clumps formed spheroids in low‐adhesion cell culture dishes (Ez‐BindShut II, Iwaki, Tokyo, Japan) with almost 100% success rate. The culture medium was serum‐free advanced DMEM/F‐12 (GIBCO, Waltham, MA) supplemented with FGF‐2 (10 ng/mL, ReproCell, Yokohama, Japan), penicillin (100 mg/mL, Wako, Osaka, Japan), streptomycin (0.1 mg/mL,Wako), gentamicin (50 *μ*g/mL, Wako), and fungizone (0.25 *μ*g/mL, GIBCO). DLD‐1 and HCT116 CRC cell lines were purchased from JCRB Cell Bank (Osaka, Japan) and RIKEN BioResource Center (Ibaraki, Japan), respectively. In order to estimate pharmacological side effects against normal tissues, human diploid fibroblasts were purchased (JCRB Cell Bank) as model cells. The origins of the fibroblasts were as follows: OUMS‐36 (embryo), TIG‐3 (fetal lung), TIG‐111 (dermal fibroblasts [DFs] from 6‐year‐old individual), TIG‐119 (DFs from 34‐year‐old individual), and TIG‐102 (DFs from 97‐year‐old individual). Adult DFs were purchased from Kurabo (Osaka, Japan). CRC cell lines and fibroblasts were maintained under supplier‐recommended culture conditions. Since the culture conditions are different between fibroblasts and CTOSs, we need to be cautious about the interpretation of data (i.e., lateral comparison between them). Indeed, we encountered some discrepancy about drug sensitivity between conventional cancer cell lines (adherent culture 12) and CTOSs (spheroid culture, in this study). However, most CTOS cells rarely attach on plastic dish and oppositely, spheroid culture of fibroblasts compromised cell survival (data not shown). Colorectal CTOSs display augmented survival through maintaining epithelial structure with E‐cadherin 16. On the other hand, the establishment of conventional cell lines (adherent culture) are much less efficient because constant cell division and autonomous survival are required for the procedure. When we choose CTOSs instead of cancer cell lines, we lose the equivalent condition (adherent culture) as a trade‐off.

### Reagents

**Figure 1:**
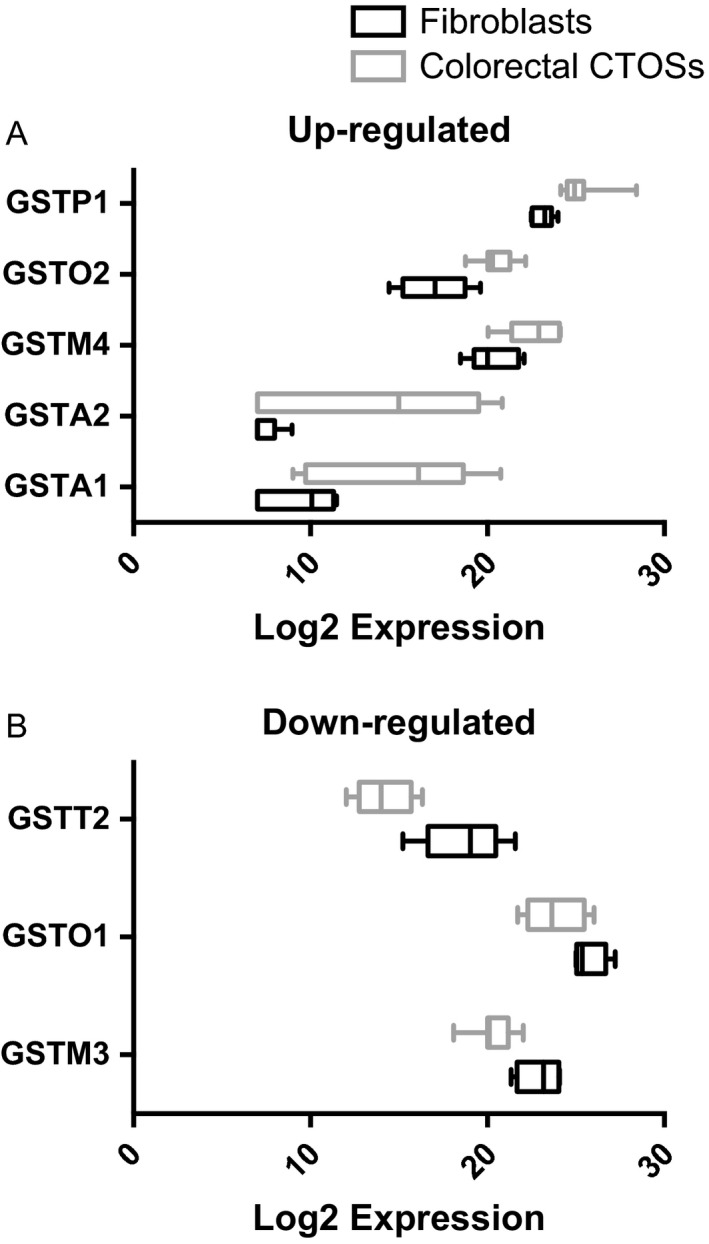
Five classes of glutathione S‐transferase are up‐regulated in colorectal cancer tissue‐originated spheroids (CTOSs), whereas three classes are down‐regulated. mRNA expression of each gene was quantified by real‐time RT‐PCR and expression level was indicated as relative Log_2_ expression. Black and gray box plots represent fibroblasts (embryonic or dermal) and CTOSs (from distinct colorectal carcinomas patients), respectively. (A) GST isoforms up‐regulated in colorectal CTOSs compared to fibroblasts. (B) GST isoforms down‐regulated in colorectal CTOSs compared to fibroblasts. GST, glutathione S‐transferase; DF, TIG111, and 119, dermal fibroblasts; TIG3 and OUMS36, embryonic fibroblasts.

The strategy for pharmacological inhibition of redox regulation systems is as follows (Fig. S1): Piperlongumine (Sigma‐Aldrich, St. Louis, MO) is a plant alkaloid 17 and selectively kills cancer cells over normal cells 12, 18. One of its molecular targets is GST*π* (encoded by the *GSTP1* gene) 12, one of the five up‐regulated isoforms in CRC CTOSs (Fig. 1). Auranofin (Sigma‐Aldrich) is a clinically approved drug for rheumatoid arthritis and considered to inhibit TXNRD 9, 11, 19. CB83 (ChemBridge, San Diego, CA) is a newly identified glucose‐6‐phosphate dehydrogenase (G6PD) inhibitor and inhibits PPP activity (G6PD is a critical enzyme for PPP) 10, 20.

### Immunocytochemistry

To validate the specificity of the above pharmacological intervention against CSCs, CRC CTOSs were dual stained with CD44v9 antibody (mouse monoclonal, Cosmobio, Tokyo, Japan) and GSTP1 (rabbit monoclonal, #138491, Abcam, Cambridge, UK) or TXNRD1 antibody (rabbit monoclonal, #167411, Abcam). CTOSs were fixed with 4% paraformaldehyde (Wako) for 30 min and rinsed with PBS, cryoprotected in 10–30% sucrose, and embedded in OCT (Sakura Finetech Japan, Tokyo, Japan). The CTOSs were cryosectioned with a thickness of 12 *μ*m. Slides were rehydrated in PBS and were reacted with the primary antibodies. Subsequently, secondary antibodies conjugated with Alexa Fluor^®^488 (#A28175, Molecular Probes, Waltham, MA) and with Alexa Fluor^®^594 (#150084, Abcam) were used to visualize primary antibodies' localizations (Fig. S2). Fluorescent photos were taken by confocal microscopy (FV10i, Olympus, Tokyo, Japan). Both GSTP1 and TXNRD1 were colocalized with CD44v9 at poorly organized mesenchymal structure of CTOSs, but not at organized epithelial structure (Fig. S2). Data indicate that both target enzymes are abundantly synthesized at CSCs.

### Flow cytometry

In order to estimate the effect of each inhibitor described above, intracellular reactive oxygen species (ROS) were measured using CellROX^™^ Deep Red reagent (Molecular Probes). Briefly, cells were seeded on 6‐well plates at 1 × 10^6^ cells per well. After cells reached 90% confluence, cells were treated with designated reagents for 1 h. CellROX^™^ Deep Red reagent was added at a final concentration of 5 *μ*mol/L to the culture medium and then incubated for 30 min at 37°C. Subsequently, the medium was removed and the cells were washed with PBS and detached using 0.25% trypsin‐EDTA. The resulting fluorescence was measured using a FACS Calibur flow cytometer (Becton Dickinson, Franklin Lakes, NJ). Menadione (100 *μ*mol/L) was added 30 min before CellROX^™^ Deep Red reagent treatment as a reference ROS inducer. Embryonic fibroblasts OUMS‐36 and TIG‐3 showed higher levels of basal ROS but displayed poor response (i.e., no ROS increase) against menadione, CB83, and piperlongumine (Fig. S3). DLD‐1 and HCT116 showed lower levels of basal ROS but displayed good response (i.e., increase of ROS) against menadione, CB83 (for HCT116), auranofin (for HCT116 and DLD‐1), and piperlongumine (for HCT116, Fig. S3). CD44v9 positivity of CTOS cells was analyzed as follows: Cancer spheroids were dispersed with serial treatments of TrypLE^™^ (20 min, GIBCO) and Accumax^™^ (10 min, Innovative Cell Technologies, San Diego, CA). Cell surface CD44v9 was stained with CD44v9 antibody (Cosmobio) as well as PE‐conjugated anti‐rat IgG2a (Becton Dickinson). The fluorescence was measured using a FACS Calibur flow cytometer (Becton Dickinson). Since CTOSs contained abundant auto‐fluorescent cells, we defined FL2^+^FL1^−^ cells as authentic PE‐positive (Fig. S4). Thus, CD44v9‐positive rate was defined as FL2^+^FL1^−^/total live cells and CD44v9‐positive fraction was further gated and split between CD44v9^low^ and CD44v9^high^ (Fig. S4), to calculate the mean fluorescent intensity of CD44v9^high^ fraction.

### Quantitative reverse transcription polymerase chain reaction

Total RNA was purified using RNAiso Plus (Takara, Osaka, Japan). Reverse transcription was performed using PrimeScript^™^ with gDNA Eraser (Takara). For quantifying the copy number of each mRNA, we used a StepOne^™^ Real‐Time PCR System (Applied Biosystems, Waltham, MA) and the Taqman^®^ method. Data were calculated according to the modified delta‐cycle of threshold (ΔC_T_) method. Normalized Log_2_ expression was defined as follows: (Log_2_ expression of a gene of interest) = 30 (arbitrary constant) + (C_T_ of 18S rRNA) − (C_T_ of a gene of interest). We used Log_2_ parameter for statistical analysis because mRNA expression follows a log‐normal distribution, whereas ΔC_T_ follows normal distribution. A gene‐specific fluorescent probe was designed using a web‐based program (https://qpcr.probefinder.com/organism.jsp). Table S1 SpreadSheet1 shows the primer sequences and corresponding fluorescent probes. Gene‐specific Taqman^®^ probes were purchased from Life Technologies, (Waltham, MA, for Cat#, see Table S1 SpreadSheet2). For PCR, Thunderbird^®^ Probe qPCR mix (Toyobo, Osaka, Japan) was used and the PCR conditions consisted of 40 cycles of two steps (95°C for 15 sec and 60°C for 50 sec).

### Determination of half‐maximal inhibitory concentration and combination index

Each drug's half‐maximal inhibitory concentration (IC_50_) was calculated based on live cell number ratios (drug treated/untreated × 100%) obtained from serial dilution of drug concentration. The length of drug exposure was 5 days. Each number of live cells was counted automatically by trypan blue exclusion (LUNA automated cell counter, Logos Biosystems, Anyang, South Korea). Cancer spheroids were dispersed with serial treatments (20 min each) of TrypLE^™^ and Accumax^™^. Thus, IC_50_ was determined based on cellular survival, but not on proliferation. We chose the survival index instead of the speed of growth because CTOSs in vitro show very slow proliferation (quiescence), which is one of the hallmarks of CSCs 2. The IC_50_ values of fibroblasts were accordingly determined based on cellular survival index during quiescence (by contact inhibition). However, it was difficult to precisely control cellular density due to gradual increase in cell size (senescence, data not shown). As a result, the variance of cellular survival was greater in fibroblasts compared to CTOSs (Fig. 6). To quantify the synergism of two distinct drugs, Combination Index (CI) was calculated 21. A CI value of about 1 (0.90–1.10) was defined as nearly additive and CI values more than (>1.10) and less than (<0.90) were defined as antagonism and synergism, respectively. IC_50_ and CI were determined using CompuSyn software (ComboSyn, Paramus, NK).

### Statistical analysis

Comparison between two groups (fibroblasts vs. CTOSs) and statistical differences in mean values (Log_2_ expression for GST isoforms, IC_50_ value, or CIs) were determined by *t* test using Excel software (Microsoft, Redmond, WA). Either Student's *t* or Welch's *t* test was used according to the homoscedasticity of two groups (determined by *F*‐test of Excel). The variance of cellular survival at different passage points (fibroblasts or CTOSs) was analyzed using SPSS (Levene's test, IBM, Armonk, NY) and Welch's *t* test was used to compare survival ratios between fibroblasts and CTOSs.

In order to judge the effect of drug treatments on gene expression, a repeated measures one‐way analysis of variance (ANOVA) was used to determine the variance of Log_2_ expression from four different groups (untreated, auranofin, CB83, and piperlongumine) using SPSS. Subsequently, the effect of each drug was determined by a paired *t* test as a *post hoc* analysis. The *P*‐values < 0.05 were defined as statistically significant.

### Protein extraction and immunoblotting

From colorectal CTOS, whole‐cell proteins were dissolved with solubilizer (7 mol/L urea, 2 mol/L thiourea, 2% Triton X‐100, Wako) and the entangled genomic DNA was sheared by sonication for 30 min. Procedures for immunoblotting have been described elsewhere 6, 10. Primary antibodies used in this study are as follows: phosphorylated NRF2 from Abcam (#76026) and Histone H3 from Cell Signaling Technologies (#4499, Danvers, MA). Phosphorylation of NRF2 is well correlated with nuclear accumulation in HCCs 6. Representative data are shown in the figures; all experiments were reproduced at least twice with different passage points.

### Lateral comparison of tumorigenesis in nude mice

Seven days after drug treatment in vitro, debris from dead cells were removed by vigorous pipetting and brief centrifugation. To enable lateral comparison of tumorigenesis between drug‐treated or ‐untreated CTOSs, survived cells were injected in left (drug‐treated) or right (untreated) side of a nude mouse, respectively (Fig. 9B). Since the counting of cell number by enzymatic dissociation compromises cell survival, the amount of spheroids were adjusted with pellet size 10. No drug was administered *in vivo* during tumor formation. About 3–4 weeks after tumor injection and a larger tumor reaches the sufficient size, mice were killed and tumors were collected and weighed.

## Results

### Differential expression of 17 classes of GST between colorectal cancer tissue‐originated spheroids and fibroblasts

To determine the pharmacological target of glutathione system, we compared the expression levels of 17 classes of GST between colorectal CTOS and fibroblasts. The normalized ΔC_T_ for *GSTA1, A2, A3, A4, A5, K1, M1, M2, M3, M4, M5, O1, O2, P1, T1, T2,* and *Z1* were determined by quantitative RT‐PCR (Table S2). Compared to fibroblasts, colorectal CTOSs showed up‐regulation of five classes of GST (*GSTA1, GSTA2, GSTM4, GSTO2,* and *GSTP1*) (Fig. 1, Table S2) and down‐regulation of three classes (*GSTM3, GSTO1,* and *GSTT2*) (Fig. 1, Table S2). Among them, we chose GST*π* (encoded by the *GSTP1* gene) as a cancer‐specific target of glutathione system.

### Target genes of NRF2, but not of TP53 were up‐regulated by pharmacological inhibition of TXNRD or GST*π* in colorectal CTOSs

**Figure 2:**
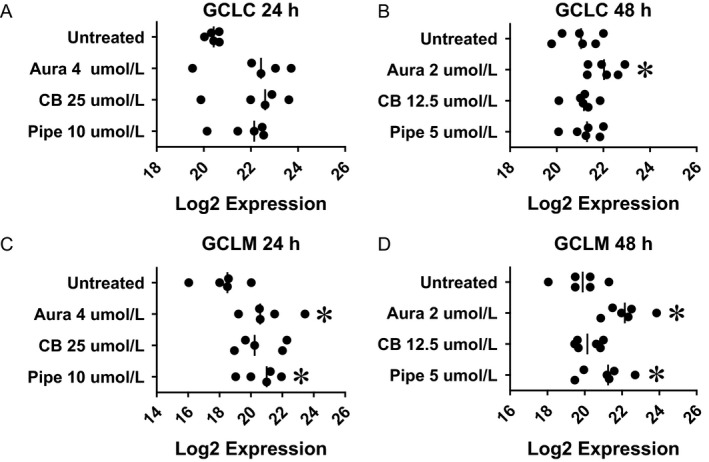
Pharmacological inhibition of redox regulation up‐regulated NRF2 target genes (glutathione system) in colorectal cancer tissue‐originated spheroids (CTOSs). Colorectal CTOSs were incubated with designated concentration of inhibitor. mRNA expression of each gene was quantified by real‐time RT‐PCR and expression level was indicated as relative Log_2_ expression. Each plot indicates Log_2_ expression of each CTOS (i.e., data from distinct patients). (A and B) Expression of GCLC. (C and D) Expression of GCLM. (A and C) Drug exposure for 24 h. (B and D) Drug exposure for 48 h. Asterisk denotes significant difference from untreated group. Aura: auranofin (TXNRD inhibitor), CB: CB83 (G6PD inhibitor), Pipe: piperlongumine (GST *π* inhibitor).

**Figure 3:**
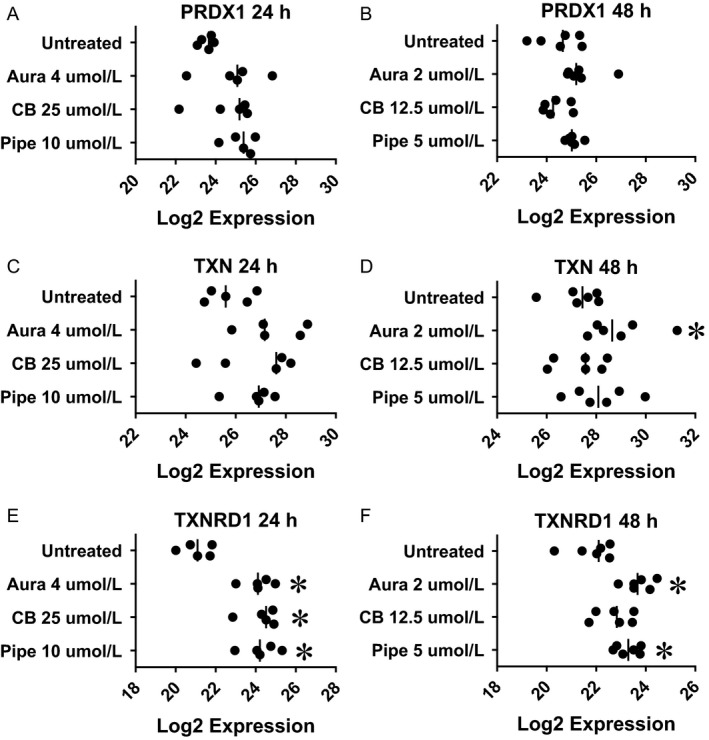
Pharmacological inhibition of redox regulation up‐regulated NRF2 target genes (thioredoxin system) in colorectal cancer tissue‐originated spheroids (CTOSs). Colorectal CTOSs were incubated with designated concentration of inhibitor. mRNA expression of each gene was quantified by real‐time RT‐PCR and expression level was indicated as relative Log_2_ expression. Each plot indicates Log_2_ expression of each CTOS (i.e., data from distinct patients). (A and B) Expression of PRDX1. (C and D) Expression of TXN. (E and F): expression of TXNRD1. (A, C, and E) Drug exposure for 24 h. (B, D, and F) Drug exposure for 48 h. Asterisk denotes significant difference from untreated group.

**Figure 4:**
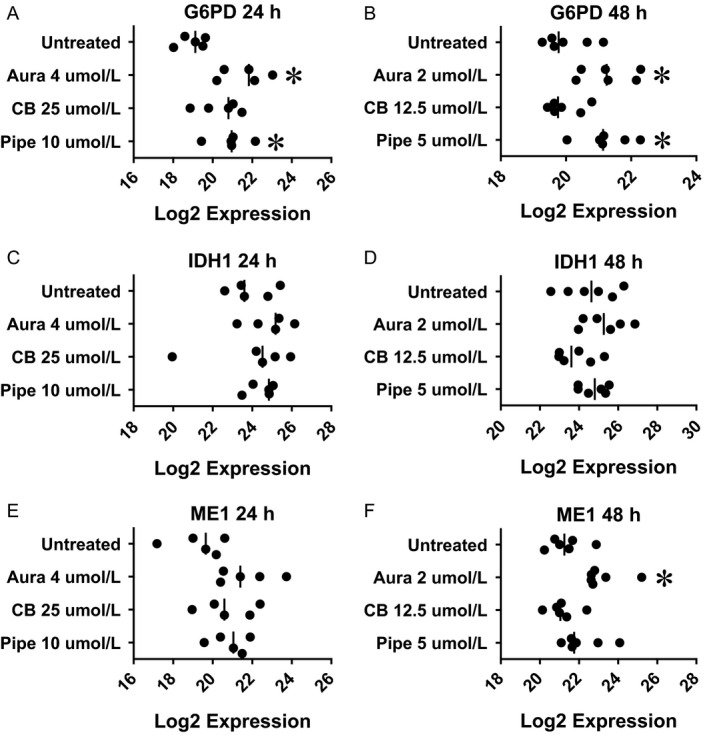
Pharmacological inhibition of redox regulation up‐regulated NRF2 target genes (NADPH production) in colorectal cancer tissue‐originated spheroids (CTOSs). Colorectal CTOSs were incubated with designated concentration of inhibitor. mRNA expression of each gene was quantified by real‐time RT‐PCR and expression level was indicated as relative Log_2_ expression. Each plot indicates Log_2_ expression of each CTOS (i.e., data from distinct patients). (A and B) Expression of G6PD. (C and D) Expression of IDH1. (E and F) Expression of ME1. (A, C, and E) Drug exposure for 24 h. (B, D, and F) Drug exposure for 48 h. Asterisk denotes significant difference from untreated group.

In order to estimate NRF2 compensation induced by pharmacological inhibition of TXNRD (auranofin), G6PD (CB83), or GST*π* (piperlongumine), we quantified gene expression of NRF2 target genes. Activation of NRF2 protein was confirmed by NRF2 phosphorylation (Fig. S5A). The Log_2_ expression for each gene with or without each inhibitor is presented in Figures 2, 3, 4.

### Glutathione system

NRF2 up‐regulates *GCLC* and *GCLM* genes, components of glutathione system. The significance of expression changes due to exposure to a higher dose (above IC_50_) for auranofin (4 *μ*mol/L), CB83 (25 *μ*mol/L), and piperlongumine (10 *μ*mol/L) for 24 h were as follows: *GCLC* (Fig. 2A, *P* = 0.055 by ANOVA) and *GCLM* (Fig. 2C, Aura: *P* = 0.045, up‐regulated, CB: *P* = 0.071, Pipe: *P* = 0.029, up‐regulated). The significance of expression changes due to exposure to a lower dose (below IC_50_) for auranofin (2 *μ*mol/L), CB83 (12.5 *μ*mol/L), and piperlongumine (5 *μ*mol/L) for 48 h were as follows: *GCLC* (Fig. 2B, Aura: *P* = 0.006, up‐regulated, CB: *P* = 0.699, Pipe: *P* = 0.17) and *GCLM* (Fig. 2D, Aura: *P* = 0.002, up‐regulated, CB: *P* = 0.479, Pipe: *P* = 0.042, up‐regulated).

### Thioredoxin system

NRF2 up‐regulates *PRDX1, TXN,* and *TXNRD1* genes, components of thioredoxin system. The significance of expression changes due to exposure to a higher dose for 24 h were as follows: *PRDX1* (Fig. 3A, *P* = 0.106 by ANOVA), *TXN* (Fig. 3C, *P* = 0.137 by ANOVA), and *TXNRD1* (Fig. 3E, Aura: *P* = 0.021, up‐regulated, CB: *P* = 0.007, up‐regulated, Pipe: *P* = 0.007, up‐regulated). The significance of expression changes due to exposure to a lower dose for 48 h were as follows: *PRDX1* (Fig. 3B, Aura: *P* = 0.066, CB: *P* = 0.766, Pipe: *P* = 0.115), *TXN* (Fig. 3D, Aura: *P* = 0.011, up‐regulated, CB: *P* = 0.851, Pipe: *P* = 0.096), and *TXNRD1* (Fig. 3F, Aura: *P* = 0.009, up‐regulated, CB: *P* = 0.154, Pipe: *P* = 0.016, up‐regulated).

### NADPH production

NRF2 up‐regulates *G6PD, IDH1,* and *ME1* genes, enzymes for NADPH production. The significance of expression changes due to exposure to a higher dose for 24 h were as follows: *G6PD* (Fig. 4A, Aura: *P* = 0.009, up‐regulated, CB: *P* = 0.099, Pipe, *P* = 0.002, up‐regulated), *IDH1* (Fig. 4C, *P* = 0.566 by ANOVA), and *ME1* (Fig. 4E, *P* = 0.0807 by ANOVA). The significance of expression changes due to exposure to a lower dose for 48 h were as follows: *G6PD* (Fig. 4B, Aura: *P* = 0.001, up‐regulated, CB: *P* = 0.659, Pipe: *P* = 0.009, up‐regulated), *IDH1* (Fig. 4D, *P* = 0.037 by ANOVA), and *ME1* (Fig. 4F, Aura: *P* = 0.017, up‐regulated, CB: *P* = 0.705, Pipe: *P* = 0.148).

### NRF2 compensation in fibroblasts

To investigate whether NRF2 compensation occurs in fibroblasts, we quantified gene induction of NRF2 target genes by auranofin (2 *μ*mol/L for 24 h) or piperlongumine (5 *μ*mol/L for 24 h). NRF2 protein was weekly phosphorylated by auranofin, but not by piperlongumine (Fig. S5A). Basal phosphorylation of NRF2 was lower in fibroblasts, compared to CTOSs (Fig. S5B). The Log_2_ expression for each gene with or without each inhibitor is presented in Fig. S6. The significance of expression changes due to the exposure were as follows: *GCLM* (Fig. S6, Aura: *P* = 0.001, up‐regulated, Pipe: *P* = 0.009, up‐regulated), *TXNRD1* (Fig. S6, Aura: *P* = 0.001, up‐regulated, Pipe: *P* = 0.439), and *G6PD* (Fig. S6, Aura: *P* = 0.001, up‐regulated, Pipe: *P* = 0.130).

### TP53 targets

TP53 up‐regulates *BBC3* (PUMA) and *CDKN1A* (P21CIP1). The significance of expression changes due to exposure to a higher dose were as follows: *BBC3* (CTOSs: Fig. S7A, *P* = 0.345 by ANOVA) and *CDKN1A* (CTOSs: Fig. S7C, *P* = 0.497 by ANOVA). The significance of expression changes due to exposure to a lower dose were as follows: *BBC3* (fibroblasts: Fig. S6, Aura: *P* = 0.031, Pipe: *P* = 0.001, up‐regulated; CTOSs: Fig. S7B, *P* = 0.757 by ANOVA) and *CDKN1A* (fibroblasts: Fig. S6, Aura: *P* = 0.036, Pipe: *P* = 0.730; CTOSs: Fig. S7D, *P* = 0.141 by ANOVA). Although we failed to detect any significant effect by drug treatment in CTOSs, ANOVA revealed individual differences among patients (i.e., some patients retained TP53 activity but the other not, *P* < 0.001) for both *BBC3* and *CDKN1A*.

### Dual inhibition of TXNRD and GST*π* displayed synergistic cell death of colorectal CTOSs but the synergism was not remarkable in fibroblasts

qRT‐PCR data suggest that auranofin or piperlongumine successfully increased intracellular ROS and the subsequent activation of NRF2 compensated the inhibition. To confirm that glutathione and thioredoxin compensate for each other through NRF2, we combined auranofin and piperlongumine at various molar ratios and quantified synergism in CTOSs and fibroblasts. The IC_50_ value for each drug and CIs are summarized in Table 1 (for details, see Table S3).

**Table 1:** Half‐maximal inhibitory concentrations (IC_50_) and Combination Indices (CI) of TXNRD inhibitor and GST*π* inhibitor

	OUMS36	TIG3	TIG111	TIG119	DF	TIG102	CTOS71	CTOS76	CTOS86	CTOS88
AuraIC50	1.79	1.96	1.75	1.17	1.59	1.09	2.21	2.83	3.02	2.34
PipeIC50	9.36	10.62	8.04	7.48	6.30	7.89	6.01	7.15	7.12	4.25
CI1:20	1.63	1.17	1.11	0.92		0.79	0.25	0.65	0.46	0.90
CI1:10	0.73	1.17	0.95	0.98	0.77	0.81	0.19	0.48	0.38	0.47
CI1:5	0.82	0.90	0.73	0.90	0.85	1.17	0.24	0.32	0.45	0.56
CI2:5	0.78	1.08	0.76	1.04	0.95	0.78	0.34	0.43		0.48

Cells were treated with serial dilution of inhibitors either alone or with combination. IC_50_ and CI were calculated based on surviving cell number (% treated/untreated and for details, see Supplementary Table S3), aided by Compusyn software. CI value around 1 (0.90–1.10) is defined as “nearly additive” and CI values more (>1.10) and less (<0.90) are defined as “antagonism” and “synergism”, respectively (see Methods). Unit for IC_50_: *μ*mol/L. CTOSs, cancer tissue‐originated spheroids; TXNRD, thioredoxin reductase.

### Ic_50_

As for single usage of each drug, CTOSs are less sensitive against auranofin than fibroblasts (Table 1, *P* = 0.0023). However, CTOSs are slightly more sensitive against piperlongumine (Table 1, *P* = 0.052).

### CIs

**Figure 5:**
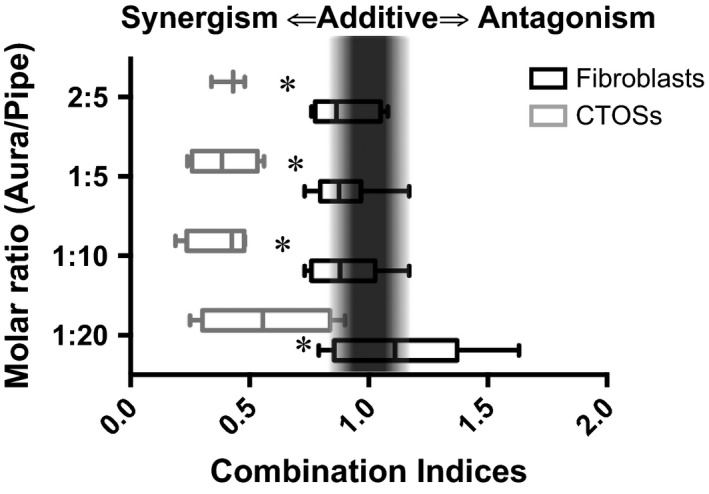
Dual inhibition of GST *π* and TXNRD results in synergistic cell death in colorectal cancer tissue‐originated spheroids (CTOSs). Cells were treated with auranofin and piperlongumine. Box plot indicates Combination Indices (CI) with molar ratios (auranofin:piperlongumine) from 1:20 to 2:5. Black and gray boxes represent fibroblasts (embryonic or dermal) and CTOSs (from distinct CRC patients), respectively. A perpendicular gray pillar is located around CI from 0.9 to 1.1, where dual inhibition displays “nearly additive” effect. CIs to the left of the pillar (<0.9) signify “synergism”. The lower the CI, the stronger the synergism. Asterisk denotes significant difference between CTOSs and fibroblasts.

Auranofin and piperlongumine displayed synergism against CTOSs (Table 1, CI < 0.7). Against fibroblasts, the dual inhibition displayed nearly additive (0.9 < CI<1.1) or at most, much weaker synergism (0.7 < CI<0.9) compared to CTOSs (Fig. 5, Table 1). Significant difference in CI was detected at molar ratio 1:20 (*P* = 0.028), 1:10 (*P* = 0.001), 1:5 (*P* = 0.001), and 2:5 (*P* = 0.001) (Fig. 5, Table 1 and Table S3).

### Ratio of surviving cells

**Figure 6:**
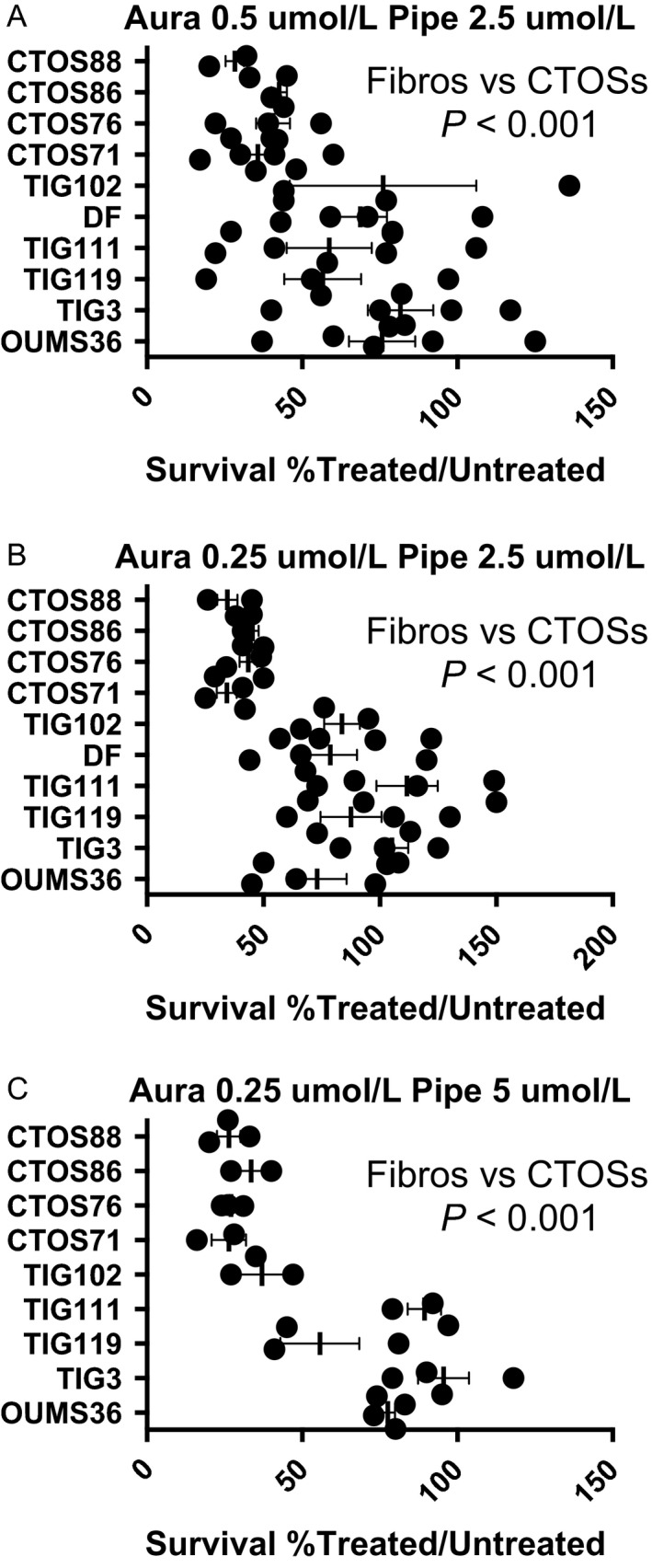
Selective killing of colorectal cancer tissue‐originated spheroids (CTOS) cells by dual inhibition of GST *π* and TXNRD. Colorectal CTOSs or fibroblasts were incubated with designated concentration of each inhibitor (molar ratio: A: 1:5, B: 1:10, and C: 1:20). Scatter plots indicate calculated percentage of survived cells (drugs treated/untreated). Experiments were repeated with different passage points. Note that colorectal CTOSs are more “sensitive” to drugs' treatment than fibroblasts are, indicating selective killing of cancer cells. Error bar represents mean ± standard error of the mean. OUMS36 and TIG3: embryonic fibroblasts, TIG119, 111, 102 and DF, dermal fibroblasts. Asterisk denotes significant difference between CTOSs and embryonic fibroblasts. TXNRD, thioredoxin reductase.

In order to ensure that the cytotoxic effect is cancer‐specific, we compared the ratio of surviving cells against three different molar ratios (Fig. 6 and Table S3). The first molar ratio was 1:5 (Aura 0.5 *μ*mol/L + Pipe 2.5 *μ*mol/L, Fig. 6A). CTOSs were more sensitive to dual inhibition than fibroblasts (Fig. 6A, *P* < 0.001 by Welch's *t* test), but the variance was larger in fibroblasts when data were taken from different passage points (probability of homoscedasticity = 0.001 by Levene's test). The second molar ratio was 1:10 (Aura 0.25 *μ*mol/L + Pipe 2.5 *μ*mol/L, Fig. 6B). CTOSs were more sensitive to dual inhibition than fibroblasts (Fig. 6A, *P* < 0.001), but the variance was larger in fibroblasts when compared to CTOSs (*P* < 0.001). The third molar ratio was 1:20 (Aura 0.25 *μ*mol/L + Pipe 5 *μ*mol/L, Fig. 6C). CTOSs were more sensitive to dual inhibition than fibroblasts (Fig. 6C, *P* < 0.001), but the variance was larger in fibroblasts when compared to CTOSs (*P* = 0.003).

### A small subpopulation of CTOS showed persistent survival against TXNRD/GST*π* dual inhibition and accumulated double‐strand DNA breaks

**Figure 7:**
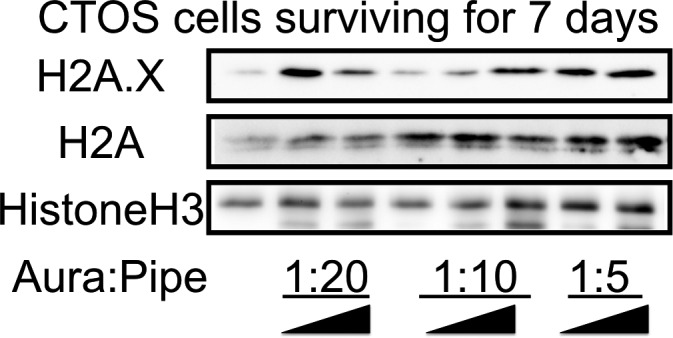
Prolonged incubation of GST *π*/TXNRD inhibitors induces drug resistance and double‐strand DNA breaks in colorectal cancer tissue‐originated spheroids (CTOS) cells. Colorectal CTOS cells were incubated with designated molar ratio and serial increment of two inhibitors for 7 days. Although majority of CTOS cells were killed during prolonged exposure to drugs, small number of cells display drug resistance. Whole‐cell proteins were extracted from such “persisters” and each well was loaded with same amount of proteins (20 *μ*g for H2A.X, 30 *μ*g for H2A, and 10 *μ*g for H3). Phosphorylation of histone H2 (H2A.X, a marker of double‐strand DNA breaks) was visualized by western blotting. Blotting for histone H2A and H3 serve as loading controls. TXNRD, thioredoxin reductase.

Comparison of IC_50_ values between fibroblasts and CTOSs indicate that drug treatment could reduce the size of the bulk tumor but could not eliminate all the cancer cells, in realistic therapeutic concentrations (e.g., Aura 0.25 *μ*mol/L + Pipe 2.5 *μ*mol/L). Indeed, we noted that a small number of CTOS cells persistently survive even after prolonged incubation with a higher dose (e.g., Aura 1 *μ*mol/L + Pipe 10 *μ*mol/L), at which most fibroblasts were eliminated. We therefore collected such persisters and examined the drugs' effect for double‐strand (ds) DNA breaks, a downstream event caused by ROS induction 22. Seven days after drug exposure, a dose‐dependent increase in H2A.X (termed also as γH2AX, phosphorylated histone H2A, a surrogate marker of dsDNA break) was observed in CTOS cells (Fig. 7). Signal of H2A.X was not apparent during 1 or 3 days of drug treatments (Fig. S8), suggesting that dsDNA breaks accumulate over time, after cells endured the rapid cell death by oxidative stress. The effect of drug exposure on total protein synthesis of histone H2A and histone H3 was negligible (Fig. 7).

### TXNRD/GST*π* dual inhibition decreased CD44v9‐positive rate of CTOSs

**Figure 8:**
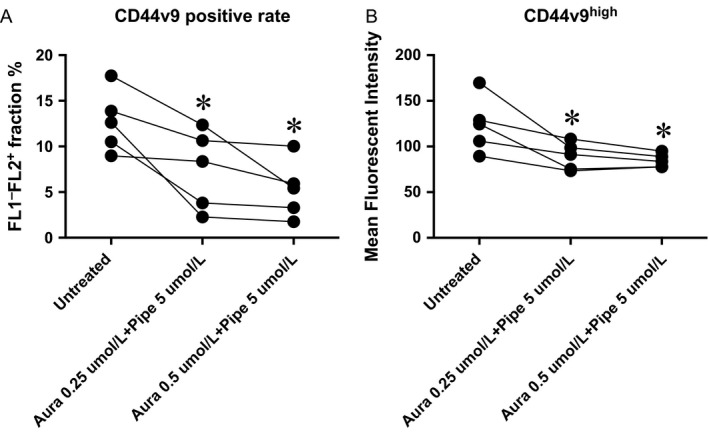
Dual inhibition of GST *π* and TXNRD decreased CD44v9 surface presentation of colorectal cancer tissue‐originated spheroids (CTOSs). Colorectal CTOSs were incubated with designated concentration of each inhibitor (molar ratio 1:20 or 1:10). Enzymatically dispersed cells were stained CD44v9 antibody and the cell surface fluorescent signal was analyzed with flow cytometry. (A) CD44v9‐positive rate (see Fig. S4 for gating strategy). (B) Mean fluorescent intensity of CD44v9^high^ population (Fig. S4). Asterisk denotes significant difference between untreated and dual inhibition (paired *t*).

Persistent survival of CTOS cells suggest the existence of remaining CSCs. To estimate the efficacy of the drug treatment against CSCs, we measured a positive rate of CD44v9 with flow cytometry. The molar ratio 1:20 (Aura 0.25 *μ*mol/L + Pipe 5 *μ*mol/L) as well as 1:10 (Aura 0.5 *μ*mol/L + Pipe 5 *μ*mol/L) decreased the CD44v9‐positive rate (Fig. 8A, *P* = 0.032 for 1:20 and *P* = 0.015 for 1:10 by paired *t*). Mean fluorescent intensity of CD44v9^high^ population was also decreased by TXNRD/GST*π* dual inhibition (Fig. 8B, *P* = 0.037 for 1:20 and *P* = 0.031 for 1:10).

### TXNRD/GST*π* dual inhibition delayed tumor formation

**Figure 9:**
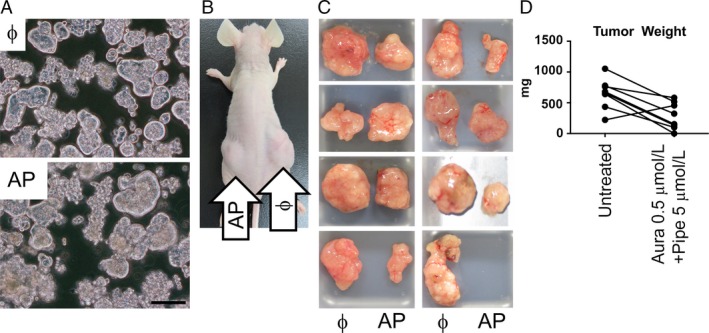
Dual inhibition of GST *π* and TXNRD delayed tumor formation and growth in vivo. Colorectal cancer tissue‐originated spheroids (CTOSs) were incubated with auranofin 0.5 *μ*mol/L + piperlongumine 5 *μ*mol/L for 7 days. (A) Drug‐resistant CTOSs retained spheroid structure (AP) similar to untreated (ϕ) CTOSs. (B) Pairwise comparison of tumor formation between untreated (ϕ) and dual inhibition (AP). (C) Collected tumors. (D) Tumor weights after collection (*P* = 0.008 by paired *t* test) Scale bar: 100 *μ*m. TXNRD, thioredoxin reductase.

After TXNRD/GST*π* dual inhibition in vitro, survived CTOS cells still retained spheroid structure (Fig. 9A). To estimate the significance of such “persisters” for residual disease, we compared tumor formation between untreated and drug‐treated (Aura 0.5 *μ*mol/L + Pipe 5 *μ*mol/L) CTOSs (Fig. 9B). While untreated CTOSs formed visible tumors with approximately 1 week, drug pretreated CTOSs did not form tumors until 2 or 3 weeks after injection. As a result, tumor weight was heavier in untreated group compared to drug‐treated group at the point of tumor collection (Fig. 9C and D, *P* = 0.008 by paired *t*,* n* = 8). In one case, the drug pretreatment abrogated tumor emergence completely (Fig. 9C).

## Discussion

One of molecular bases of radiation or conventional chemotherapy is thought to be increase in intracellular ROS 23. In general, cancer cells produce abundant ROS owing to oncogene activation and proliferation, simultaneously maximizing ROS protection by NRF2 activation 24. In addition, cancer cells obtain energy without the aid of mitochondria, one of the major sources of intracellular ROS (Warburg effect 25, 26). On top of that, CSCs protect themselves through maintaining cellular redox potential lower than their differentiated progeny 22. Thus, cancer cells routinely spend majority of their resources for redox regulation, rendering them vulnerable against further ROS insults, possibly explaining why radiation or chemotherapy is effective despite the apparent nonspecificity 23. Therefore, inhibiting this ROS protection system is one of the promising approaches to eradicate CSCs 9.

Using patient‐derived colorectal CTOSs and their xenografts 10, we made several discoveries. First, five GST isoforms (*GSTA1, A2, M4, O2,* and *P1*) are up‐regulated in colorectal CTOSs relative to fibroblasts. Second, pharmacological inhibition of GST*π* (*GSTP1*) or TXNRD up‐regulated NRF2 target genes, but not TP53 targets, in CTOSs. Third, dual inhibition of GST*π* and TXNRD synergistically killed CTOS cells and the synergism was observed preferentially in CTOSs and not in fibroblasts. Fourth, a small subpopulation of CTOSs persistently survived the dual inhibition and a marker of double‐strand DNA breaks was increased in such population. Fifth, the dual inhibition successfully decreased surface presentation of CD44v9. Sixth and finally, the dual inhibition delayed the tumor emergence *in vivo* but the “persisters” still retain tumorigenicity.

We chose one of the GST isoforms as the pharmacological target of glutathione system. GST has 17 isoforms and we have described the differential expression profile between colorectal CTOSs and fibroblasts (Fig. 1). Piperlongumine selectively kills cancer cells and increase intracellular ROS 12. Since GST*π* is one of the molecular targets of piperlongumine, we expected that selective killing would be reproduced in colorectal CTOS cells. However, the difference in IC_50_ (CTOSs vs. fibroblasts) of piperlongumine was moderate (*P* = 0.052, Table S3), indicating that the pharmacological window of selective killing is not wide enough. This could be due to the augmented feedback regulation of NRF2 because piperlongumine up‐regulated the genes for thioredoxin system and NADPH production in colorectal CTOSs (Figs. 3, 4 and S5). The reason for discrepancy between the previous report and ours remains clear; it could be due to difference in cell culture conditions (see Methods). On the other hand, we found that TXNRD inhibition is more toxic against fibroblasts than against CTOSs (Tables 1 and S3). In CTOSs, auranofin up‐regulated the genes for glutathione system and NADPH production (Figs. 2 and 4), suggesting that CTOSs are more resilient against thioredoxin inhibition due to NRF2 feedback. Pharmacological inhibition of critical enzyme for PPP (G6PD) had negligible effects in terms of NRF2 feedback, with the exception of TXNRD1 induction at 24 h. Previously, we found that G6PD inhibitor CB83 reproducibly decreased NADPH/NADP^+^ ratios, but failed to alter GSH/GSSG ratios in CRC cell lines 10. While the compensation mechanisms remain unknown, other NADPH production pathway 27 could be involved in NRF2‐independent manner.

Based on the above observations, we hypothesized that mutually compensating glutathione and thioredoxin systems should be blocked simultaneously to eliminate cancer cells. Recently Harris et al. reported that combined inhibition of both systems leads to synergistic cancer cell death *in vitro* and *in vivo* 11. They used sulfasalazine, buthionine sulfoximine (BSO), and auranofin to inhibit cystine/glutamate transporter, glutamate–cysteine ligase (GCL), and TXNRD, respectively. The study was the first proof‐of‐concept and we have subsequently designed experiments to confirm the feasibility. We adopted a “Combination Index” to quantify the synergism of two drugs 21 and used several fibroblasts to estimate side effects against normal cells. Auranofin and piperlongumine exhibited stronger synergism in colorectal CTOSs than in fibroblasts at molar ratios 2:5, 1:5, 1:10, and 1:20 (Fig. 5). There exist drug concentration windows wherein substantial numbers of CTOSs, and not fibroblasts, were killed (Fig. 6). Although Harris et al. did not compare the IC_50_ and the synergism between cancer and normal cells 11, combining BSO and auranofin could induce intolerable side effects. This is because, unlike GST, GCL has a few isoforms and pharmacological inhibition could damage normal cells almost equally. In this regard, targeting more cancer‐specific glutathione system subunits (i.e., GSTs) is more feasible therapeutic option. Indeed, piperlongumine activated NRF2 in CTOSs but not in fibroblasts, suggesting that GST*π* inhibition is more detrimental against cancer cells due to *GSTP1* up‐regulation (Figs. 1, S5 and S6). We verified that combining piperlongumine and auranofin is a promising alternative, but piperlongumine has additional off‐target effects 18, 28. In addition, we do not exclude the possibility that other up‐regulated GST isoforms (*α*1, *α*2, *μ*4, or *ο*2, Fig. 1) are superior target to GST*π*. Developing more specific GST*π* inhibitor or inhibitors against other CRC‐specific GSTs could further refine the specificity.

Previously, we reported that NRF2 is activated and up‐regulates PPP enzymes in HCC specimens 6. More recently, we identified the p62 phosphorylation as a potential cause of constitutive activation of NRF2 29. Considering the fact that NRF2 is frequently activated during carcinogenesis by point mutations and other epigenetic alteration 30, 31, it was rather surprising that colorectal CTOSs displayed apparently physiological NRF2 response against oxidative stress (this study). We observed that basal NRF2 phosphorylation was higher in CTOSs compared to fibroblasts (Fig. S5), and up‐regulated GST isoforms are known NRF2 target genes (Fig. 1). Data collectively indicate the possibility that CRC cells increase basal level of NRF2 activity but still retain the potential of further up‐regulation. Thus, regulation of NRF2 could not be explained by binary switch (i.e., on or off), but be explained by gradual increase for fine‐tuning of the redox potential.

Finally, we propose the existence of another mechanism of drug resistance against redox inhibition. While we refined the specificity and effectiveness of redox inhibition against CSCs, it was difficult to completely eliminate them. A small subpopulation of CTOSs persistently survived GST*π*/TXNRD dual inhibition despite accumulated double‐strand DNA breaks (H2A.X, Figs. 7 and S8). Importantly, we confirmed that the dual inhibition lowered CD44v9 surface presentation (Fig. 8) and delayed the tumor emergence in nude mouse (Fig. 9). However, survived “persisters” still retained lesser but significant tumorigenicity, indicating that decreased CD44v9 does not necessarily mean the complete remission. Therefore, GST*π*/TXNRD dual inhibition preferentially killed CSCs but nevertheless the treatment was not enough to eradicate them completely. We selectively used CTOS cells especially malignant in terms of nude mouse tumorigenesis and CD44v9 presentation. Such experimental system could mimic *in vivo* phenomena that distant metastasized cancer cells survive ROS insults induced by radiation or chemotherapy (pan‐resistance). How such “persisters” survive intensive ROS insults should be addressed in the future.

## Conclusions

Colorectal CTOSs up‐regulated five GST isoforms, but inhibition of one of them (GST*π*) displayed modest cancer‐specific cytotoxicity. Inhibition of either GST*π* or TXNRD up‐regulates NRF2 target genes to compensate the other. Dual inhibition results in synergistic cell death in CTOSs, expanding the window of “selective killing of cancer cells”. The dual inhibition successfully decreased CD44v9 surface presentation, and delayed the tumor emergence in vivo. However, a small number of “persisters” survived the synergistic cell death despite accumulated DNA damage and retained lesser but significant tumorigenicity.

## Conflict of Interest

The authors declare that they have no conflicts of interest.

## Supporting information


**Figure S1.** Schematic diagram of pharmacological inhibition of redox regulation.
**Figure S2.** CD44v9 is colocalized with GSTP1 and TXNRD1.
**Figure S3.** Intracellular ROS is higher in fibroblasts than in CRC cell lines. Inhibitors of redox regulation increase the ROS in CRC cell lines.
**Figure S4.** Strategy of gating CD44v9^positive^ population and defining CD44v9^low^ or CD44v9^high^.
**Figure S5.** Pharmacological inhibition of redox regulation induced NRF2 phosphorylation by inhibitors in colorectal CTOSs.
**Figure S6.** Pharmacological inhibition of redox regulation up‐regulated NRF2 target genes, but not TP53 target genes, in human fibroblasts.
**Figure S7.** Pharmacological inhibition of redox regulation failed to alter expression of TP53 target genes in colorectal cancer CTOSs.
**Figure S8.** Prolonged incubation of GST*π*/TXNRD inhibitors induces double‐strand DNA breaks in survived colorectal CTOS cells.

**Table S1.** Sequences of oligos and fluorescent probes used in this study.

**Table S2.** Expression profiles of 17 classes of GST.

**Table S3.** Inhibition of GST*π* and/or TXNRD on cellular survival of embryonic fibroblasts.
