# Supplementary material for: Dual pharmacological inhibition of glutathione and thioredoxin systems synergizes to kill colorectal carcinoma stem cells
Source: Cancer Med. 2016 Aug 3;5(9):2544–57. doi: 10.1002/cam4.844 (PMC5055185; doi:10.1002/cam4.844)
Supplement: Supplementary file 1 — Figure S1. Schematic diagram of pharmacological inhibition of redox regulation. Figure S2. CD44v9 is colocalized with GSTP1 and TXNRD1. Figure S3. Intracellular ROS is higher in fibroblasts than in CRC cell lines. Inhibitors of redox regulation increase the ROS in CRC cell lines. Figure S4. Strategy of gating CD44v9positive population and defining CD44v9low or CD44v9high. Figure S5. Pharmacological inhibition of redox regulation induced NRF2 phosphorylation by inhibitors in colorectal CTOSs. Figure S6. Pharmacological inhibition of redox regulation up‐regulated NRF2 target genes, but not TP53 target genes, in human fibroblasts. Figure S7. Pharmacological inhibition of redox regulation failed to alter expression of TP53 target genes in colorectal cancer CTOSs. Figure S8. Prolonged incubation of GSTπ/TXNRD inhibitors induces double‐strand DNA breaks in survived colorectal CTOS cells. [file CAM4-5-2544-s001.docx]

Supplementary Figure1


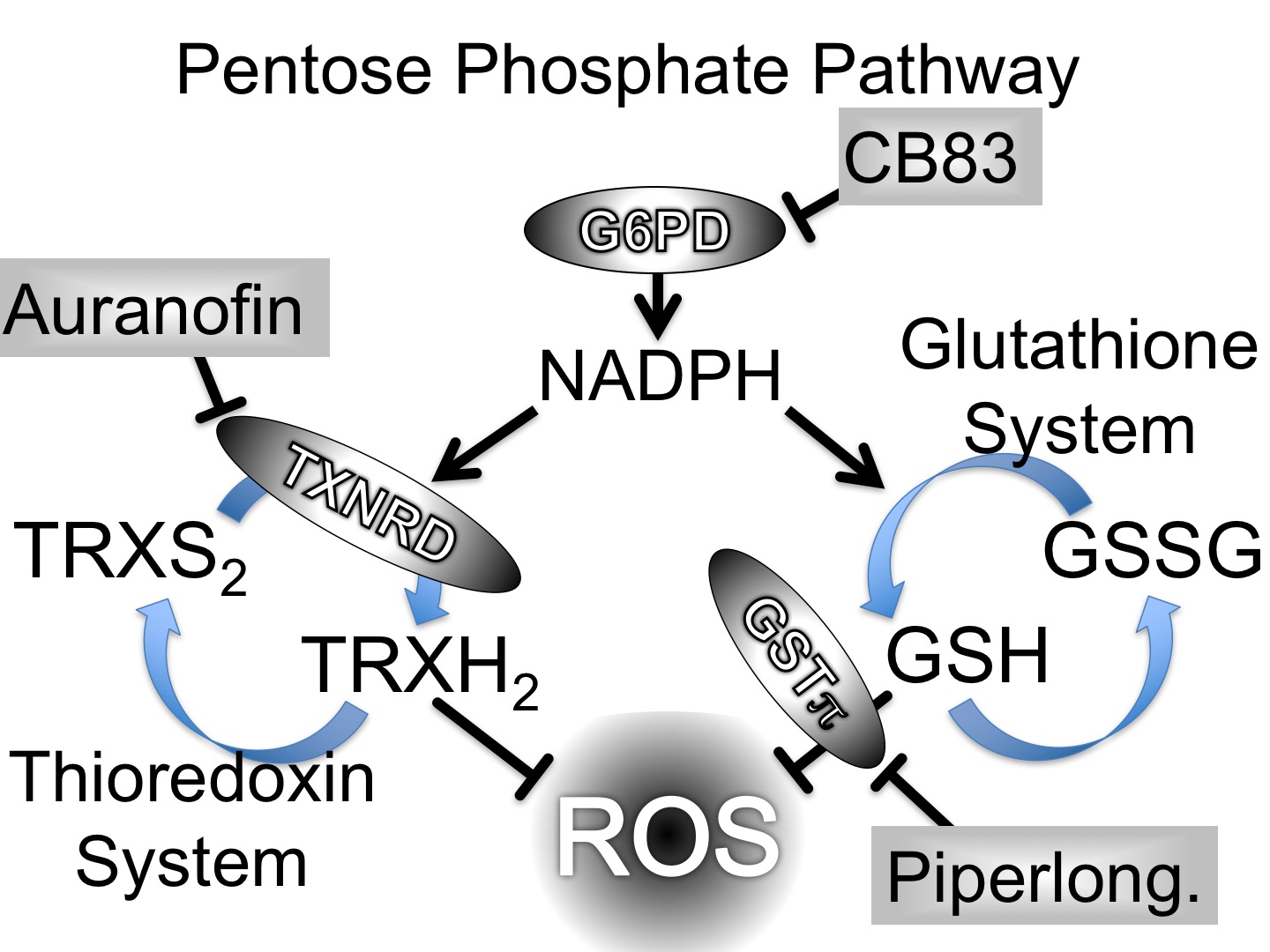


**FigureS1**

**Schematic diagram of pharmacological inhibition of redox regulation**

Reduced glutathione (GSH) and reduced thioredoxin (TRXH_2_) reduce reactive oxygen species (ROS) and are themselves oxidized to GSSG and TRXS_2_, respectively (antioxidant reactions). For two recycling reactions (GSSG→GSH and TRXS_2_→TRXH_2_), reducing power of NADPH is required. G6PD is a critical enzyme of pentose phosphate pathway (PPP), which is augmented and a major source of NADPH in cancer cells. In this study, we used inhibitors auranofin (TXNRD inhibitor), piperlongumine (GSTπ inhibitor) and CB83 (G6PD inhibitor) to disrupt the redox regulation of colorectal cancer spheroids.


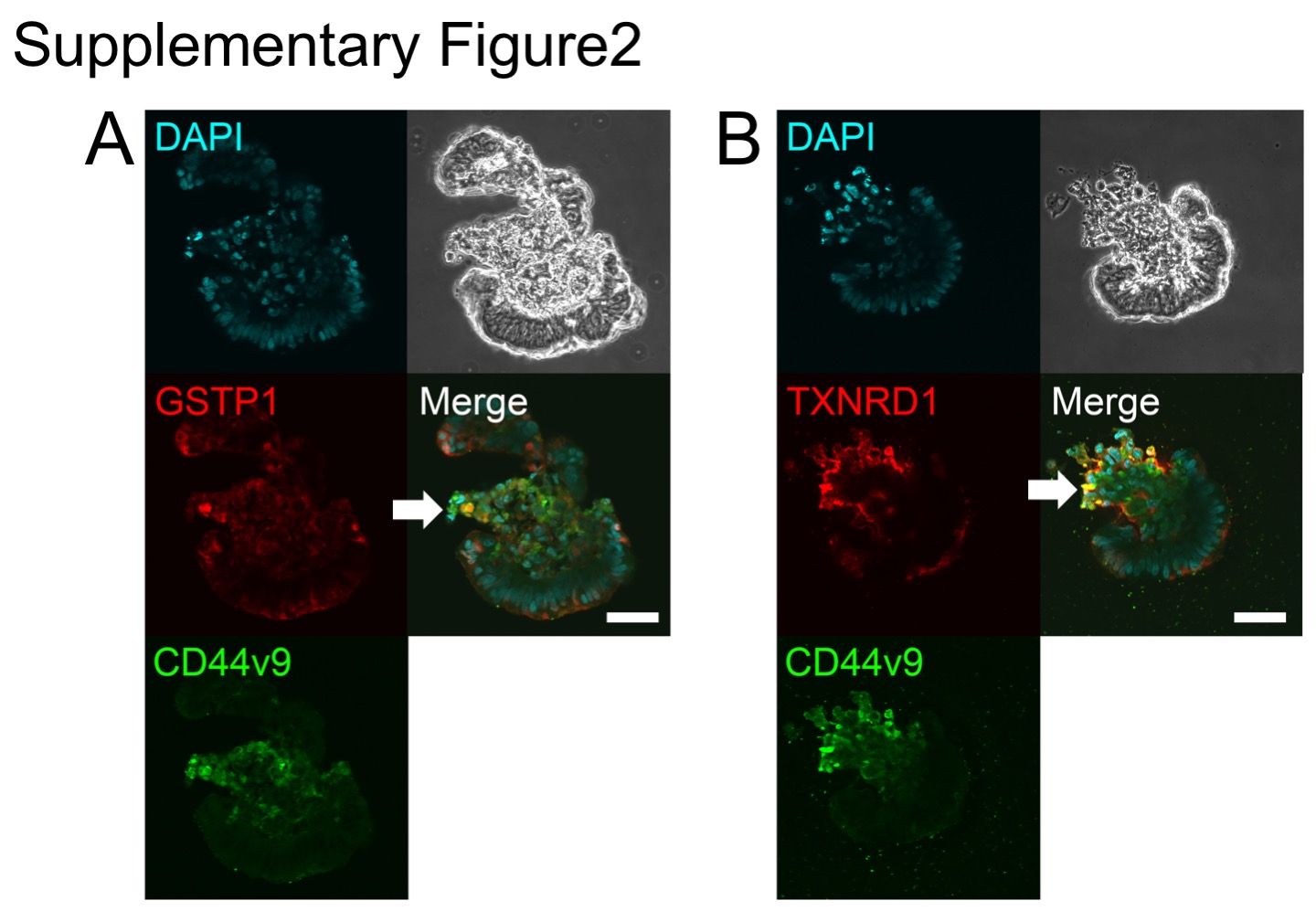


**FigureS2**

**CD44v9 is co-localized with GSTP1 and TXNRD1.**

A: Cryosection of colorectal CTOSs was dual stained with anti-CD44v9 antibody (green) as well as GSTP1 (red) and visualized by fluorescent secondary antibodies. B: A: Cryosection of colorectal CTOSs was dual stained with anti-CD44v9 antibody (green) as well as TXNRD1 (red) and visualized by fluorescent secondary antibodies. Cell nuclei were counter-stained with DAPI. CD44v9 was not localized at well-organized epithelial structure, but was localized at poorly-organized mesenchymal structure. Note that CD44v9 and GSTP1/TXNRD1 were co-localized each other (white arrows). Scale bars: 50μm


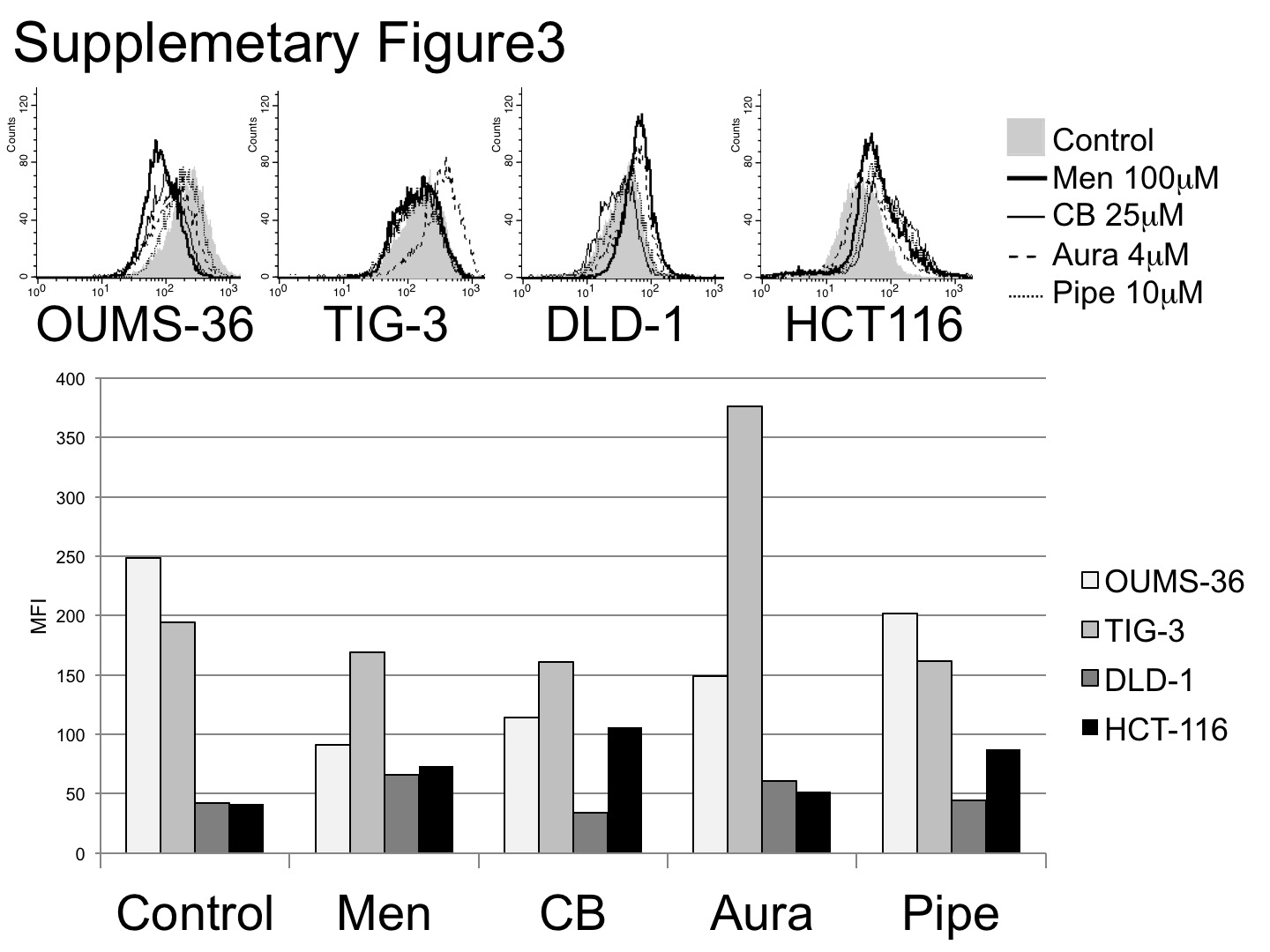


**FigureS3**

**Intracellular ROS is higher in fibroblasts than in CRC cell lines. Inhibitors of redox regulation increase the ROS in CRC cell lines.**

One hour after each drug treatment, cells were stained with CellROX DeepRed and intracellular ROS were quantified with FACS. Upper: histogram of fluorescent signal is represented. Lower. representative mean fluorescent intensity scores are shown. Note that without drug treatment (Control), intracellular ROS is higher in OUMS36 and TIG3 (embryonic fibroblasts) but lower in DLD-1 and HCT116 (CRC cell lines). ROS in DLD-1 was increased by auranofin. ROS in HCT116 was increased by all the inhibitors tested. Menadione was used as a reference chemical to increase ROS.

**
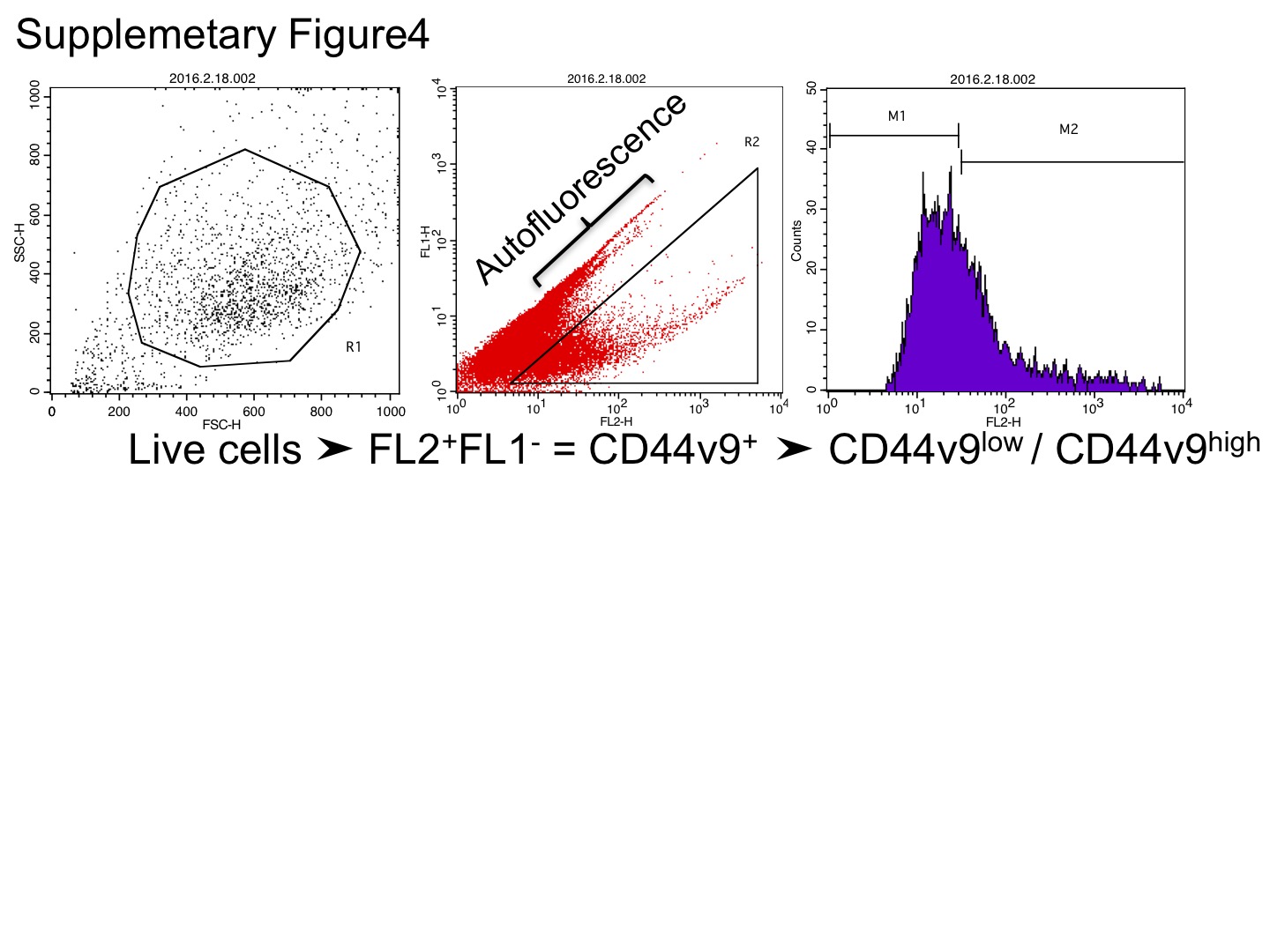
FigureS4**

**Strategy of gating CD44v9^positive^ population and defining CD44v9^low^ or CD44v9^high^.**

Three days after each drug treatment, CTOS cells were enzymatically dispersed and stained with CD44v9 antibody (visualized by PE conjugated secondary antibody). FL2^+^FL1^-^ population was defined as CD44v9^positive^ and we confirmed the negligible number of cells were positive in such criteria in isotype control (data not shown). CD44v9^positive^ cells were further split into CD44v9^low^ and CD44v9^high^ population.


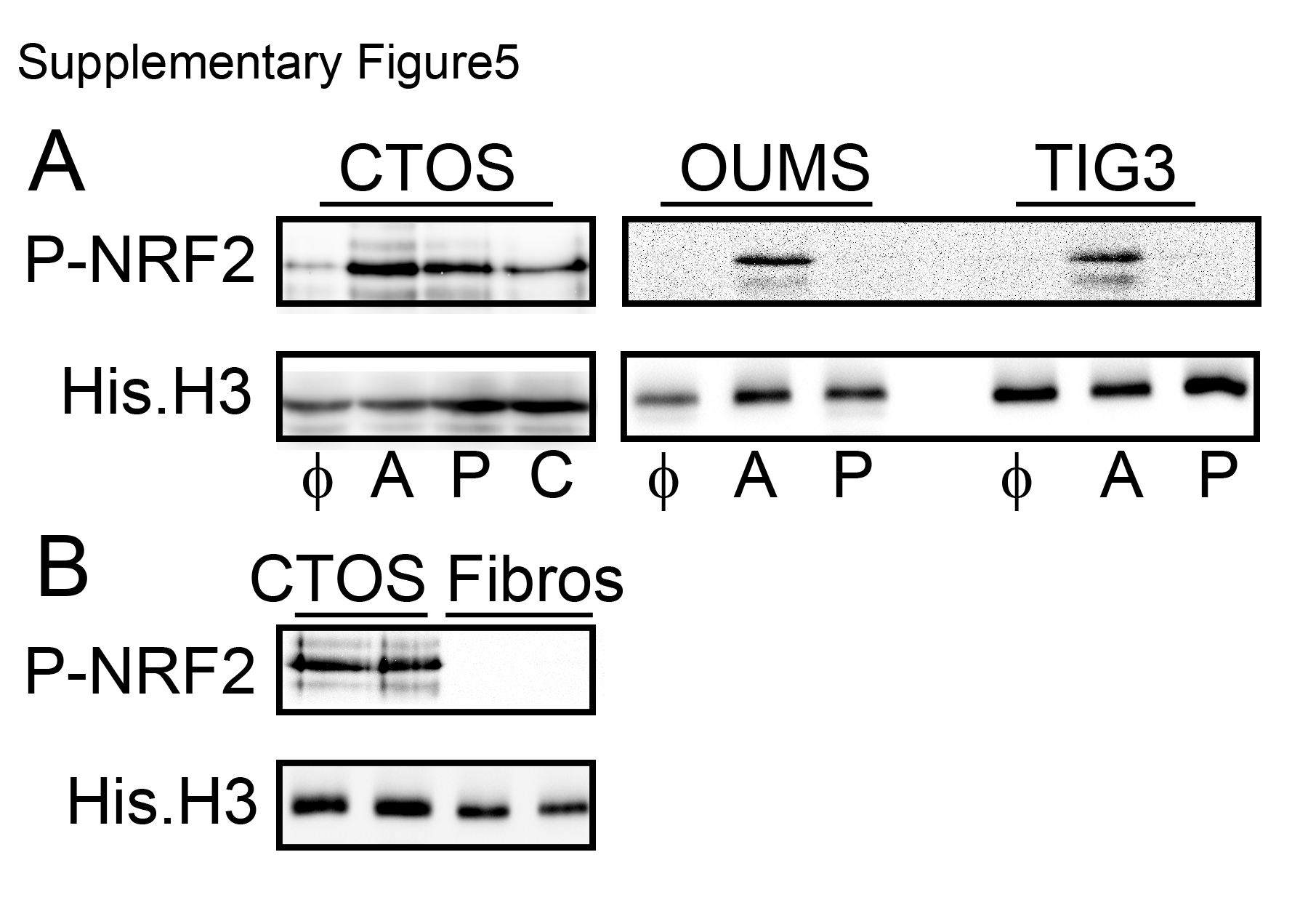


**FigureS5**

**Pharmacological inhibition of redox regulation induced NRF2 phosphorylation by inhibitors in colorectal CTOSs**

Colorectal CTOSs were incubated with each inhibitor for 24 hours. Whole cell proteins were extracted and each well was loaded with same amount of proteins (40 μg for NRF2 and 10 μg for H3). Phosphorylation of NRF2 was measured by western blotting. Blotting for histone H3 serves as loading control. A: NRF2 was efficiently phosphorylated by Auranofin, Piperlongumine or CB83 in colorectal CTOSs. In contrast, NRF2 was less efficiently phosphorylated in embryonic fibroblasts (OUMS and TIG3) by the same concentration of Auranofin. The effect of Piperlongmine was almost negligible. φ: untreated, A: Auranofin 2μM, P: Piperlongumine 5μM, C: CB83 12.5 μM B: Basal level of phosphorylation was different between colorectal CTOSs and embryonic fibroblasts.

**
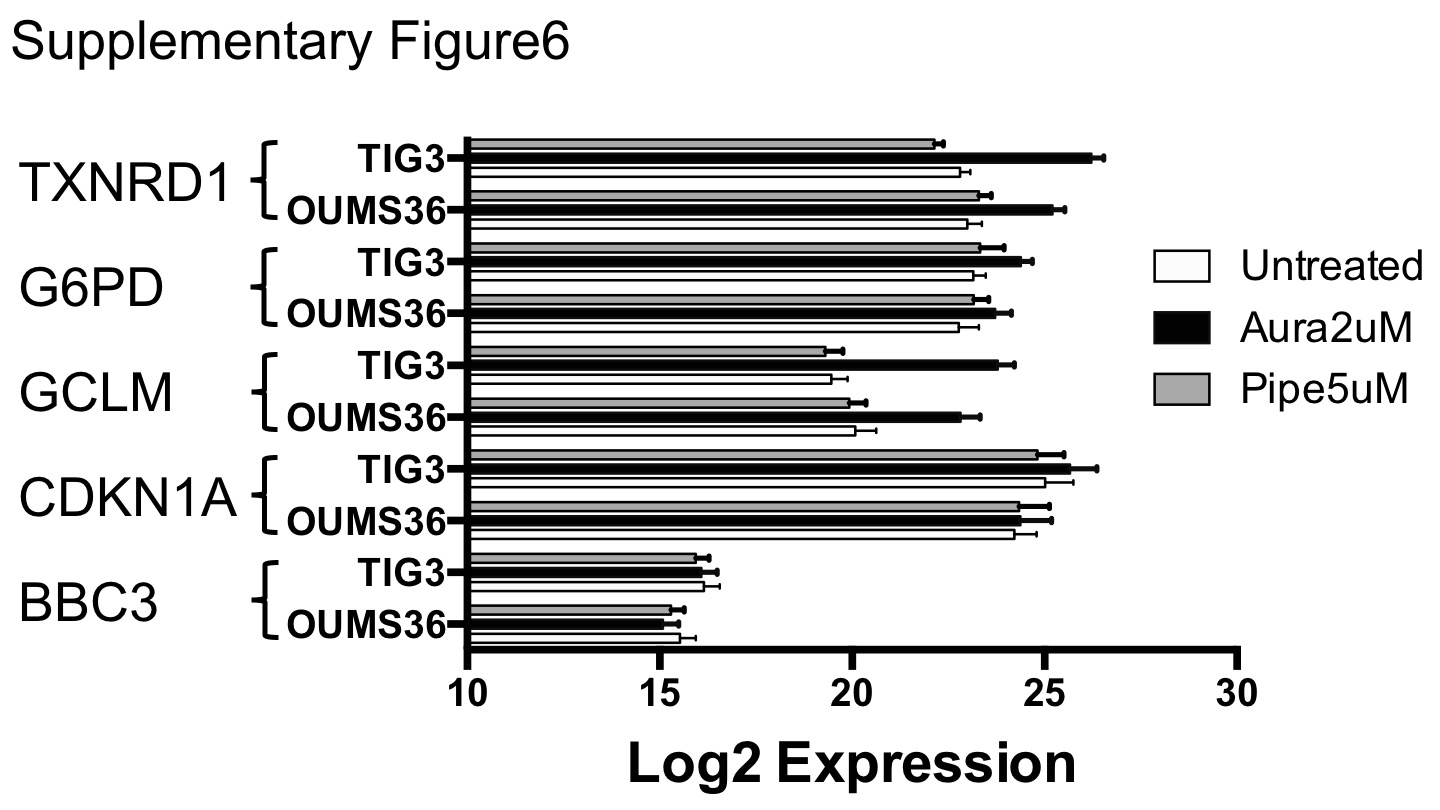
**

**FigureS6**

**Pharmacological inhibition of redox regulation up-regulated NRF2 target genes, but not TP53 target genes, in human fibroblasts**

Human embryonic fibroblasts OUMS36 or TIG-3 was incubated with designated concentration of inhibitor for 24 hours. mRNA expression of each gene was quantified by real-time RT-PCR and expression level was indicated as relative Log_2_ expression. Each bar indicates mean value of Log_2_ expression (n=3 at different passage points). Error bar indicates standard deviation. P-values of difference are described in Results.

**
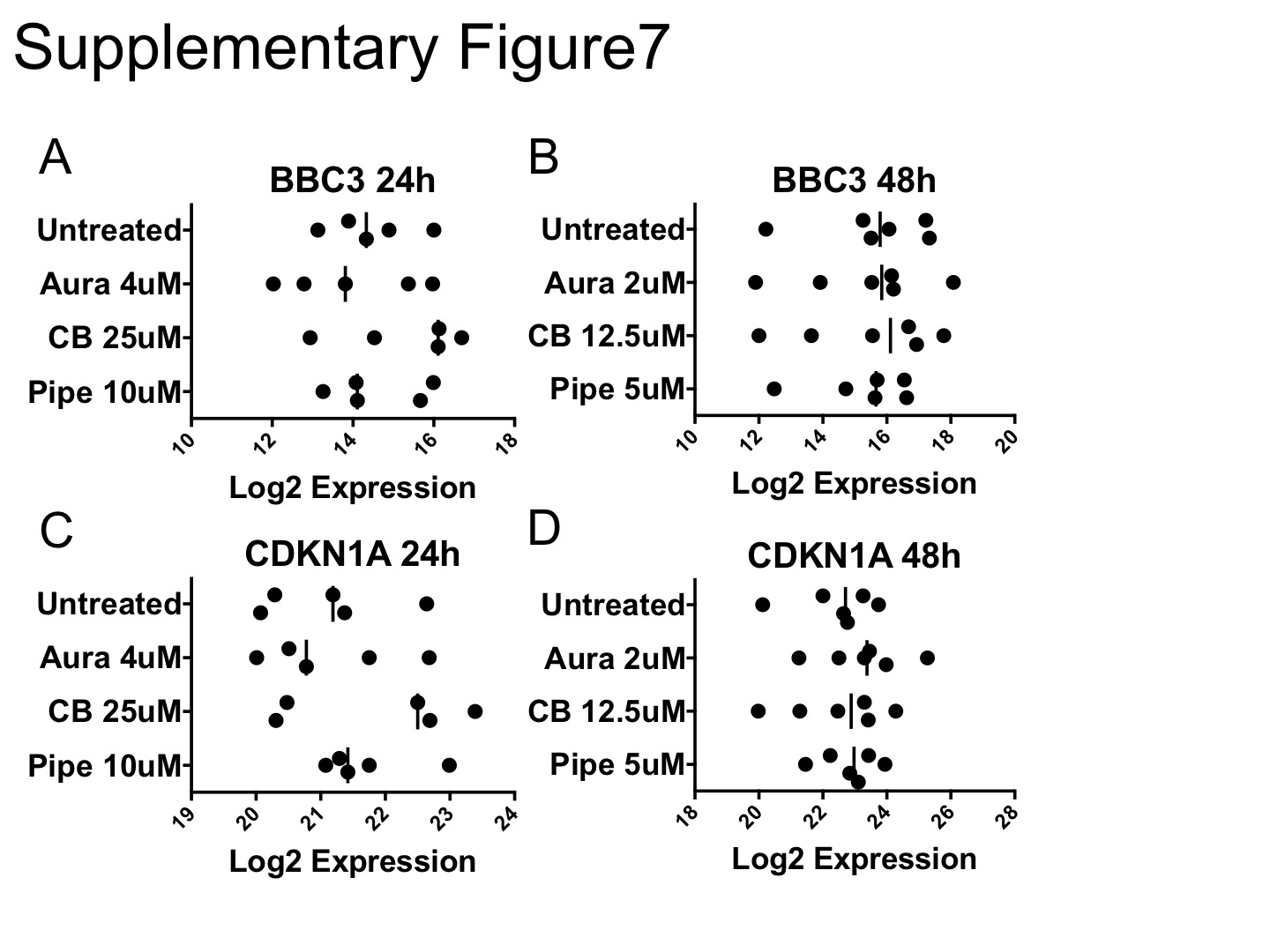
**

**FigureS7**

**Pharmacological inhibition of redox regulation failed to alter expression of TP53 target genes in colorectal cancer CTOSs**

Colorectal CTOSs were incubated with designated concentration of inhibitor. mRNA expression of each gene was quantified by real-time RT-PCR and expression level was indicated as relative Log_2_ expression. Each plot indicates Log_2_ expression of each CTOS (i.e. data from distinct patients). A and B: Expression of BBC3 (PUMA). C and D: Expression of CDKN1A (P21CIP1). A and C: Drug exposure for 24 hours. B and D: Drug exposure for 48 hours.


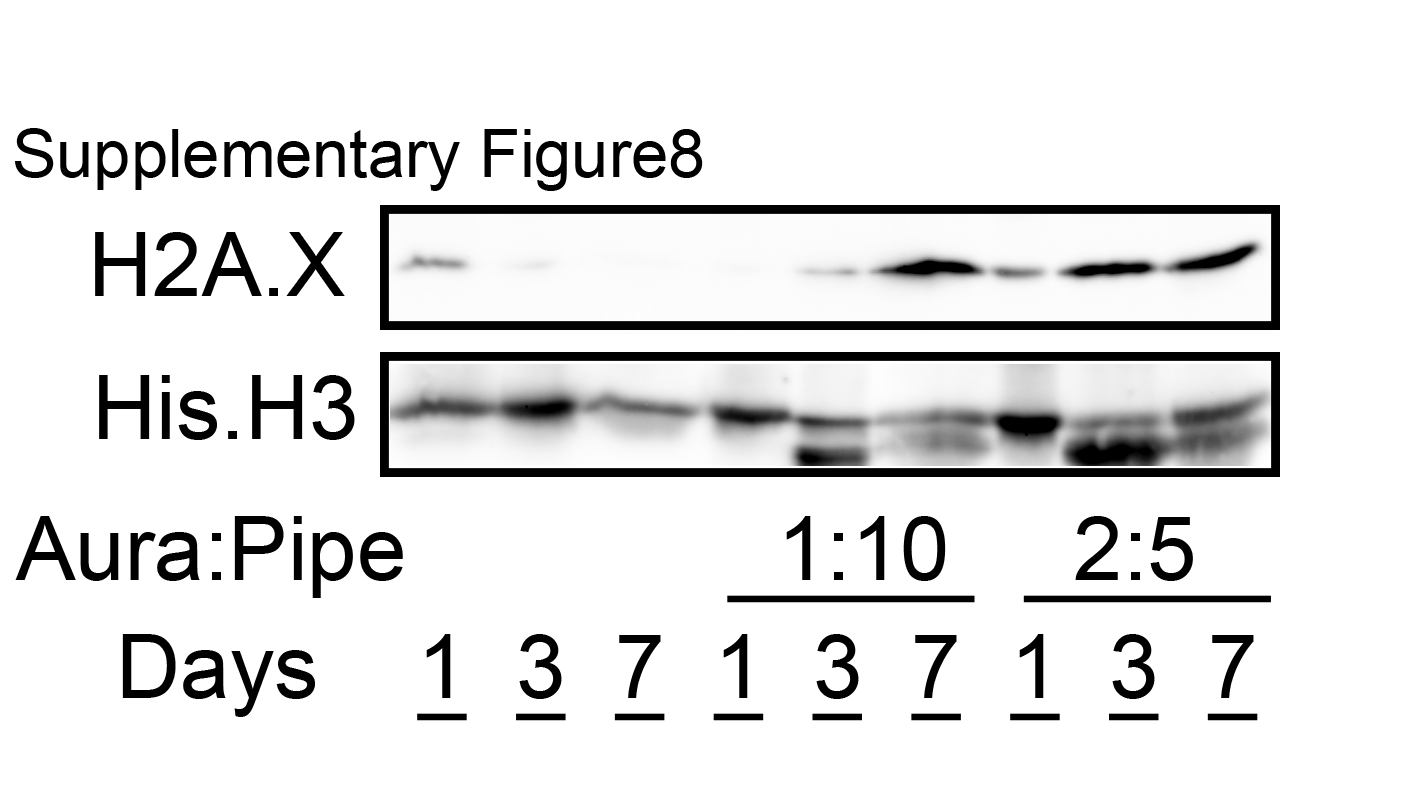


**FigureS8**

**Prolonged incubation of GSTπ/TXNRD inhibitors induces double-strand DNA breaks in survived Colorectal CTOS cells**

Colorectal CTOS cells were incubated with designated concentration (μM) and the time course (treatment for one, three or seven days) was followed. Although majority of CTOS cells were killed during prolonged exposure to drugs, small number of cells display drug resistance. Whole cell proteins were extracted from such "persisters" and each well was loaded with same amount of proteins (20 μg for H2A.X and 10 μg for H3). Phosphorylation of Histone H2 (H2A.X, a marker of double-strand DNA breaks) was visualized by western blotting. Blotting for histone H3 serves as loading controls.
